# PINTnet: construction of condition-specific pathway interaction network by computing shortest paths on weighted PPI

**DOI:** 10.1186/s12918-017-0387-3

**Published:** 2017-03-14

**Authors:** Ji Hwan Moon, Sangsoo Lim, Kyuri Jo, Sangseon Lee, Seokjun Seo, Sun Kim

**Affiliations:** 1 Interdisciplinary Program in Bioinformatics, Seoul National University, Seoul, Republic of Korea; 2 Department of Computer Science & Engineering, Seoul National University, Seoul, Republic of Korea; 3 Bioinformatics Institute, Seoul National University, Seoul, Republic of Korea

**Keywords:** Pathway interaction, Closeness centrality, Shortest path, Protein-protein interaction, Gene expression

## Abstract

**Background:**

Identifying perturbed pathways in a given condition is crucial in understanding biological phenomena. In addition to identifying perturbed pathways individually, pathway analysis should consider interactions among pathways. Currently available pathway interaction prediction methods are based on the existence of overlapping genes between pathways, protein-protein interaction (PPI) or functional similarities. However, these approaches just consider the pathways as a set of genes, thus they do not take account of topological features. In addition, most of the existing approaches do not handle the explicit gene expression quantity information that is routinely measured by RNA-sequecing.

**Results:**

To overcome these technical issues, we developed a new pathway interaction network construction method using PPI, closeness centrality and shortest paths. We tested our approach on three different high-throughput RNA-seq data sets: pregnant mice data to reveal the role of serotonin on beta cell mass, bone-metastatic breast cancer data and autoimmune thyroiditis data to study the role of IFN- *α*. Our approach successfully identified the pathways reported in the original papers. For the pathways that are not directly mentioned in the original papers, we were able to find evidences of pathway interactions by the literature search. Our method outperformed two existing approaches, overlapping gene-based approach (OGB) and protein-protein interaction-based approach (PB), in experiments with the three data sets.

**Conclusion:**

Our results show that PINTnet successfully identified condition-specific perturbed pathways and the interactions between the pathways. We believe that our method will be very useful in characterizing biological mechanisms at the pathway level. PINTnet is available at http://biohealth.snu.ac.kr/software/PINTnet/.

## Background

### The importance of finding perturbed interaction between pathways

Identifying perturbed pathways in a given condition is crucial in understanding biological phenomena. Over-representation analysis (ORA) [1], gene set enrichment analysis (GSEA) [2–4], signaling pathway impact analysis (SPIA) [5] and EnrichNet [6] are widely used approaches to identify such pathways. These approaches detect activated pathways and rank the pathways in terms of their own activation scores or statistical tests. However, pathways usually function in a coordinated and cooperative fashion [7–9]. Thus understanding interactions or crosstalk between pathways becomes as important as identifying perturbed single pathway.

### Currently available methods of pathway interaction network

There are several approaches to find pathway interactions and construct a pathway interaction network. The first and simplest one is to consider the shared components such as genes or proteins between pathways. It assumes that shared genes may mediate interactions and predicts such interactions by testing the significance of the overlapping genes between pathways using hypergeometric test such as Fisher’s exact test [10]. The second approach is to estimate interactions using protein-protein interaction (PPI) information. This approach assumes that any two interacting pathways may have more edges connected in PPI than expected. An approach used topological information in PPIs connecting pathways to construct a pathway interaction network [11]. An improved method for finding active PPIs between pathways was also studied [12]. Since statistical significance cannot guarantee biological significance and vice versa, the aforementioned approaches are likely to miss biologically meaningful interactions. To address this issue, a function-based approach was suggested [13]. The approach estimated pathway interaction based on Gene Ontology (GO) similarities.

### Motivation

Though the previously mentioned methods suggested an insight on how to predict interactions between pathways, the methods are based mainly on testing differential gene expression. None of these methods use the explicit quantity of gene expression. Therefore, the methods are not able to identify the subtle but important changes in gene expression. Moreover, many of the methods do not take into account the topological features, treating the pathways just as a set of genes. Recently, a path-based approach was studied [14] but it does not use transcriptomic data to predict condition-specific interactions, limiting itself to finding merely static interactions between pathways.

Here, we propose a new **p**athway **int**eraction **net**work construction method (PINTnet). The summary of our method are:

1. The interactions between pathways are represented by the subnetworks that are constructed considering two topological features: closeness centrality and shortest path.
2. Shortest paths on the subnetworks are computed based on an assumption that pathway interactions occur by a series of spontaneous reactions among genes belonging to the pathways.
3. The explicit quantity of gene expression is used to measure the activation status of pathway interactions.
4. The flow of the changes in expression is weighted. Higher weight is given to any edge between genes when the edge connects differentially expressed genes (DEGs).

## Methods

**Fig. 1:**
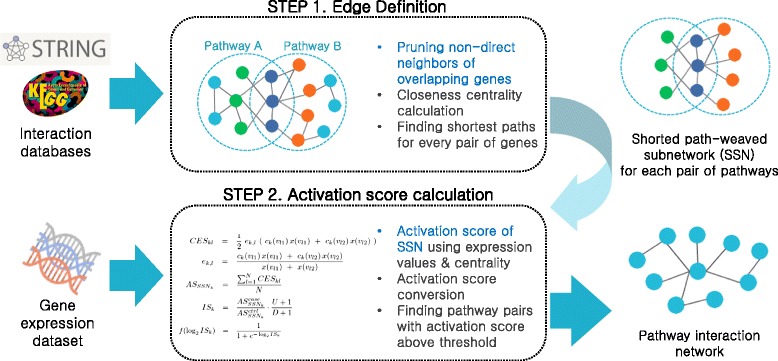
Overview of our method

In this section, we describe the process of how PINTnet measures the activation status of the interactions and constructs the pathway interaction network including the preprocess steps in detail. The overview of the method is depicted in Fig. 1.

### Preparation of PPI and pathway information

We collected protein-protein interaction data from STRING (ver.9.1) [15] and pathway data from KEGG (Release 73.1) [16]. To integrate these two independent information, we selected the genes in both datasets and edges in the pathways are augmented by bringing in edges in STRING. There are several main categories of pathways in KEGG. These are metabolism, genetic information processing, environmental information processing, cellular processes, organismal systems and human diseases. We excluded pathways in metabolism category because the metabolic pathways focus on the metabolic products of cells and are not well represented in PPI [17].

### Defining edges in the pathway network

Defining edges between two pathways is the key issue in constructing a pathway interaction network. Below are the steps for defining edges.

#### Step 1: Subnetwork construction on each pathway pair

We constructed a subnetwork for every possible pair of pathways. To do so, we used two criteria for the pathways to be paired: whether the two pathways have at least one overlapping gene and whether the two pathways have at least one gene connected to the overlapping genes via PPI. We defined every pair of two pathways as a possible pair only when the two pathways satisfied the both criteria. Then, for every possible pair, a subnetwork was constructed using PPI involving the two pathways.

#### Step 2: Closeness centrality calculation

For each subnetwork generated above, we calculated closeness centrality of all the genes within. The centrality was to evaluate the degree of a node to be central in a given network, by taking a reciprocal of an average shortest path length to all the nodes within a network from the source. The shorter the average shortest path length of a node, the closer to 1 the closeness centrality of the corresponding node is, otherwise, closer to zero. In this way, genes reflect the topological importance of themselves concerning all possible neighbor nodes within a given subnetwork.

#### Step 3: Shortest path computation

After calculating the closeness centrality, we pruned the genes that are not direct neighbors to overlapping genes in subnetworks. Then, we computed the shortest paths. Given two pathways *A* and *B*, let the genes in *A* as *A*_*genes*_={*a*_1_,*a*_2_,…,*a*_*m*_} and the genes in *B* as *B*_*genes*_={*b*_1_,*b*_2_,…,*b*_*n*_} where *m* is the number of genes in *A* and *n* is the number of genes in *B*. The shortest paths were computed for every pair of genes *a*_*i*_ and *b*_*j*_ where 1≤*i*≤*m*, 1≤*j*≤*n* and both *a*_*i*_ and *b*_*j*_ are the direct neighbor genes to the overlapping genes. The shortest paths must pass through any overlapping gene of the two pathways.

#### Step 4: Constructing shortest path-weaved subnetwork

**Fig. 2:**
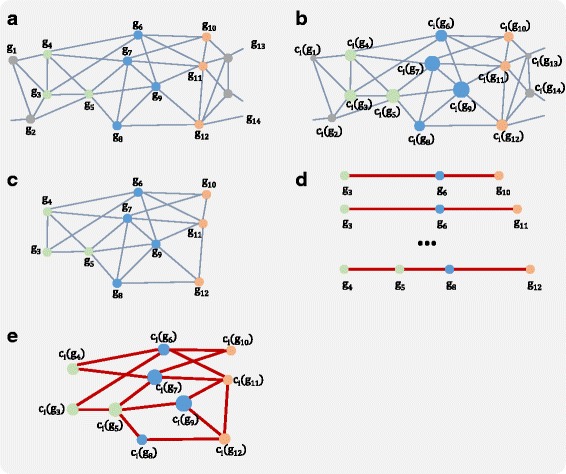
Constructing a shortest path-weaved subnetwork. *g* indicates genes. *c*_*i*_ indicates the closeness centrality of a gene of subnetwork *i*. Overlapping genes are colored in *blue*, the direct neighbors of the overlapping genes belonging to pathway A are colored in *green* and the direct neighbor genes belonging to pathway B are colored in *orange*. The others are colored in *gray*. **a** A subnetwork of pathway A and pathway B **b** Closeness centrality is calculated for every gene in the subnetwork. The node size represents the closeness centrality of the node. **c** The genes that are not direct neighbors to overlapping genes are pruned. **d** Shortest paths are computed. **e** The shortest paths are weaved to construct a shortest path-weaved subnetwork

Finally, we weaved the shortest paths and constructed shortest path-weaved subnetworks (SSN). We conjectured that the pathway interaction occurs by the rapid and spontaneous flow of biological signal or interaction through topologically important genes. This concept is realized in our method by computing shortest path in the weighted subnetworks in terms of closeness centrality. The SSN thus is the network that connects the topologically important genes using the shortest paths. The overview of these steps is depicted in Fig. 2.

### Measuring activation status of pathway interaction

Measuring the activation status of biological systems or networks is technically difficult. For example, we may want to compute the average expression level of all genes in a network as the activation status of the network. However, this computation completely ignores topological features. A recent work [18] demonstrated that the identification and measurement of subsystems by using both PPI and pathway information were effective in prognosis of breast cancer survival by defining activation status of network edges. We incorporated the approach to calculate the activation status of interaction between pathways. To measure the activation status of each SSN, PINTnet firstly calculates a co-expression score (CES) of each edge of the SSN using the following equations:

1 $$\begin{array}{@{}rcl@{}} {CES}_{kl}&=&\frac{1}{2} e_{k,l} \left(c_{k}\left(v_{l1}\right)\,x\left(v_{l1}\right) + c_{k}\left(v_{l2}\right)\,x\left(v_{l2}\right)\right) \end{array} $$

2 $$\begin{array}{@{}rcl@{}} e_{k,l}&=&\frac{c_{k}\left(v_{l1}\right)\,x\left(v_{l1}\right)\ +\ c_{k}\left(v_{l2}\right)\,x\left(v_{l2}\right)}{x\left(v_{l1}\right)\ +\ x\left(v_{l2}\right)} \end{array} $$

where *k* is the index of SSN constructed from each pathway pair, *l* is the index of an edge, *v*_*l*1_ and *v*_*l*2_ are two genes connected by the edge *l*, *c*_*k*_(*v*) is the closeness centrality of a gene *v* in *S* *S* *N*_*k*_ and *x*(*v*) is the expression level of a gene *v*. *e*_*k*,*l*_ is the condition-specific edge centrality of edge *l* in *S* *S* *N*_*k*_. After measuring the co-expression score for every edge in *S* *S* *N*_*k*_, PINTnet takes the average of the summation of the scores as the activation score (AS) and that is:

3 $$\begin{array}{@{}rcl@{}} {AS}_{{SSN}_{k}}&=&\frac{\sum_{l=1}^{N}{CES}_{kl}}{N} \end{array} $$

where *N* is the total number of edges in *S* *S* *N*_*k*_ and *l* is the index of an edge. PINTnet then calculates the ratio of ${AS}_{{SSN}_{k}}\phantom {\dot {i}\!}$ for the case and the control data so it can reflect the activity of the pathway interaction in a comparative manner between case and control.

Computing DEGs is a simple but effective approach for detecting perturbed pathways and even signaling impacts in the pathways in a given condition. However, DEGs are widely interspersed and are not connected in the networks or pathways. To utilize DEG information, we applied the ratio of DEGs that are connected by edges as a weight. A higher interaction score is assigned for more DEG connections. In this step, PINTnet simply calculates the fold change of expression level of each gene to define DEGs and the default threshold is log20.5 as used in other studies that used RNA-seq data [19–21]. The equation is as follows:

4 $$\begin{array}{@{}rcl@{}} {IS}_{k}&=&\frac{{AS}_{{SSN}_{k}}^{case}}{{AS}_{{SSN}_{k}}^{ctrl}}\cdot\frac{U+1}{D+1} \end{array} $$

where *k* is the index of *SSN*, *I* *S*_*k*_ is the interaction score of *S* *S* *N*_*k*_, ${AS}_{{SSN}_{k}}^{case}$ is the activation score of *S* *S* *N*_*k*_ of the case data, ${AS}_{{SSN}_{k}}^{ctrl}$ is the activation score of *S* *S* *N*_*k*_ of the control data, *U* is the number of connected up-regulated DEGs of the case data compared to the control data and *D* is the number of connected down-regulated DEGs of the case data compared to the control data. When PINTnet calculates the fold change, the cutoff value of 1.0 for the expression level is used to prevent noise such as extremely high fold change due to the comparison between small numbers. The cutoff value was set based on other studies [22, 23]. In addition, genes that are overlapped among multiple pathways can cause false positives. A study reported this issue and proposed an approach of ruling out the overlapping genes when determining perturbed pathways [24]. We tried to attenuate the effect of those genes by dividing the expression level by the number of pathways that the genes belong to, so that it could be naturally considered in calculating the ratio of connected DEGs.

### Pathway interaction network construction

After measuring the activation status of all pairs of pathways and obtaining the interaction score, PINTnet converts the interaction score using the sigmoid function [25]. It is to convert the scores to a value in the range between 0 and 1, so a constant cutoff value can be applied uniformly to all SSNs to construct the pathway interaction network using the only pairs satisfying the cutoff. PINTnet uses log2-transformed interaction scores as the input values of the sigmoid function. The equation of the function is as follows:

5 $$\begin{array}{@{}rcl@{}} f\left(\log_{2}{IS}_{k}\right)&=&\frac{1}{1+e^{-\log_{2}{IS}_{k}}} \end{array} $$

After the interaction score is converted, a pathway interaction network is constructed with the edges between pathways when the interaction score of edges satisfies the cutoff value. We empirically determined the cutoff value by testing PINTnet on various data from other biological researches.

## Results

To evaluate the performance of PINTnet, we used three different high-throughput RNA-seq datasets in Gene Expression Omnibus (GEO). Cytoscape was used to visualize the networks [26]. The test datasets are summarized in Table 1. For the evaluation, we investigated the evidences for every edge that connected the pathways reported in the original papers through the literature search and established the evaluation criteria for the performance of PINTnet and two existing pathway interaction network construction methods, overlapping gene-based approach (OGB) and PPI-based approach (PB), were used for the performance comparison. The details of the approaches are described in Performance comparison to other methods section.

**Table 1:** The description of three datasets

Name	Title	Accession No.
Dataset1	Serotonin regulates pancreatic beta cell mass during pregnancy	GSE21860
Dataset2	ABL kinases promote breast cancer osteolytic metastasis	GSE69125
Dataset3	IFN- *α* mediates the development of autoimmunity	GSE25115

### Data description

*Dataset1* is the data that measured the gene expression levels of pregnant mice to reveal how serotonin regulates pancreatic beta cell mass during pregnancy [27]. The authors compared the global gene expression patterns in islets from nonpregnant and pregnant female mice by the high-throughput sequencing to identify the genes potentially involved in regulating maternal beta cell mass. They stated that *Tph1* and *Tph2* were the genes most markedly induced during pregnancy. These two genes encode two isoforms of tryptophan hydroxylase, the rate-limiting enzyme in the synthesis of serotonin, 5-HT. The authors also reported that beta cells share a common gene expression program and the ability to synthesize, store and secrete serotonin with serotonergic neurons.

*Dataset2* is the data generated by a study investigating how ABL kinases promote breast cancer osteolytic metastasis [28]. Bone is one of the primary sites where breast cancer metastasizes and 70% of deaths of breast cancer is caused by bone metastases. The authors evaluated the result of single- or double-knockdown of ABL1 and ABL2 in breast cancer cells using RNA-seq analysis to reveal the signaling pathways required for ABL kinases-dependent bone metastasis. They carried out GSEA to identify which pathways were affected by ABL kinases in metastatic breast cancer cells. They reported that Jak-STAT signaling pathway, Hippo signaling pathway, cytokine-cytokine interaction and bone metastasis were enriched in the control group compared to ABL1/ABL2 knockdown group.

*Dataset3* is from a study that used thyroiditis as a model to reveal how IFN- *α* plays a pivotal role in auto immunity [29]. The authors generated transgenic mice overexpressing IFN- *α* in the thyroid and performed RNA-seq analysis. The transgenic mice showed upregulation of pathways such as antigen presentation pathway, interferon signaling, complement system, apoptosis, pattern recognition receptors and RAR activation.

### Evaluation criteria

#### Dataset1

It is well known that nutrient requirements by the fetus incur change in the maternal metabolism during pregnancy. Nutrient flow to the fetus is maintained by increasing insulin resistance. The resistance may cause maternal hyperglycemia but the glucose level is maintained by the expansion of beta cells driven by prolactin and placental lactogen [30–32]. Failures in this response raise the risk of being diagnosed with gestational diabetes mellitus [33]. Serotonin is a regulator of insulin secretion and co-localized with insulin in granules of pancreatic *β*-cells. A lack of serotonin in *β*-cells can lead to reduced insulin secretion [34]. Also, it is known that prolactin has direct effects on increasing insulin secretion [35–37] and is closely related to diabetes [38].

#### Dataset2

Ras signaling pathway is the pathway which *ABL1* and *ABL2* belong to and it is known that Ras signaling pathway activation is implicated in breast cancer invasion and growth [39]. Thus the downstream of Ras signaling is considered to be a potential target against osteolytic breast cancer metastasis [40]. MAPK signaling pathway is known to be implicated in cancer-induced bone pain [41]. In addition, it is known that p38 MAPK is important in maturation and synthesis of osteoclasts [42, 43]. Wnt signaling pathway is one of the pathways dysregulated in human breast cancer and it was reported that the activity of Wnt signaling in breast cancer is significantly higher than that in bulk cancer cells [44]. Upregulation of Wnt signaling pathway has been reported to lead to increased metastasis including bone metastasis from breast cancer [45, 46]. TGF- *β* signaling pathway was reported to be important for the development of osteolytic bone metastases by numerous studies [47]. Proteoglycans participate in the control of bone tumor development and bone metastases dissemination [48]. A high sensitivity to PI3K-Akt signaling pathway characterizes triple-negative breast cancer metastasis to bone [49]. Hippo signaling pathway deregulation in breast cancer bone metastasis has been suggested that YAP and TAZ activity was increased in metastatic breast cancer [50]. In addition to the individual functions of the pathways, interactions between the pathways are reported by various studies [51–59].

#### Dataset3

It is known that Toll-like receptor signaling pathway plays an important role in autoimmunity including thyroid autoimmunity [60, 61]. Also, antigen presentation, complement system, apoptosis and pattern recognition receptors are known to involve in thyroid autoimmunity [62–65].

### Performance comparison to other methods

To compare the performance of PINTnet to other approaches, we implemented the overlapping gene-based approach (OGB) and the PPI-based approach (PB) and ran three methods including ours on the three test datasets in the previous sections.

#### Overlapping gene-based approach

This method is a two-step approach. In the first step, the activation status of each pathway was calculated using Fisher’s exact test with a contingency table dealing with two parameters: one is whether a gene is a DEG or not and the other is whether a gene belongs to the pathway or not. Then, in the second step, the significance of edges among pathways was evaluated using Fisher’s exact test. The significance of pathways and the edges among the pathways were determined at a *p*-value of 0.05 or less. The significant edges were used to construct a pathway network.

#### PPI-based approach

For PPI-based approach, we implemented the simple version of the approach since no executable code is available. We implemented the approach based on the hypothesis that the more interactions may guarantee the higher probability of interaction. To do this, we calculated the empirical *p*-values for every possible pair of pathways to find significantly interacting pathway pairs by shuffling the original PPI network 1,000 times, counting the number of round when the number of shuffled PPI edges between pathways was bigger than or equal to that of the original PPI edges and dividing the number by the number of round, in this case, 1,000. Then we adjusted the *p*-values using Bonferroni correction and took the edges with the *p*-value less than 0.05 as the significantly interacting edges. Connecting the edges, we constructed a template network and calculated active PPIs on the network using the datasets.

Running the approach on the test datasets, we observed that too many nodes and edges were connected even though multiple testing correction was performed using the Bonferroni correction. For example, there were 271 nodes and 12,264 edges for *dataset1*. It seemed almost impossible to determine which pathways and interactions between the pathways were important in the given conditions. Thus we did not include this approach for the performance comparison.

#### Comparison results

**Fig. 3:**
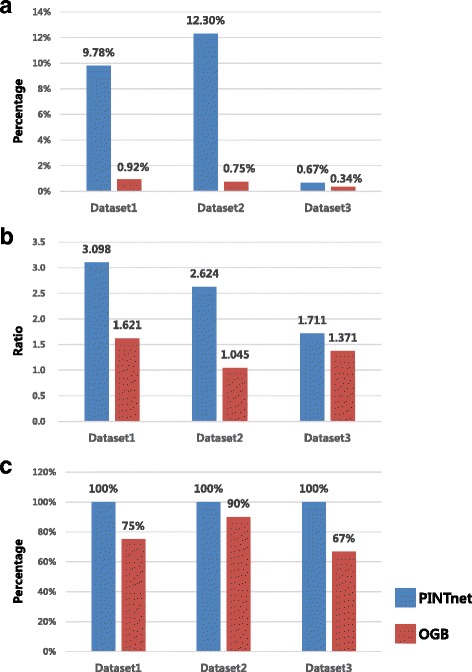
Comparison results. **a** is the percentage of the number of evidence-supported edges against the number of all edges in the pathway interaction network. PINTnet outperformed OGB in identifying the edges connected by the evidence-supported pathways. **b** is the ratio of the average degree of the evidence-supported pathways and that of all pathways in the pathway interaction network. The evidence-supported pathways had more edges when detected by PINTnet than detected by OGB. **c** is the percentage of how many evidence-supported pathways are found in the pathway interaction network

We compared the performance of the approaches based on the biological evidences found by the literature search and organized in Evaluation criteria section. The following criteria were used for the quantitative measure of the performance. The first criterion was the interactions between the pathways. We calculated the percentage of the number of the evidence-supported edges between the evidence-supported pathways against the total number of edges in the network. It was to measure how successfully the approaches connected the correct edges supported by evidence. The second criterion was the degree of pathways. We calculated the ratio of the average degree of the evidence-supported pathways against that of all pathways in the network. It was to measure how the approaches placed the important pathways as hubs in the central position of the network. The last criterion was to see how successfully the approaches rescued the correct pathways. We calculated the percentage that how many evidence-supported pathways were rescued. We confirmed that PINTnet surpasses other methods from the results described in the following paragraphs and the comparison results are shown in Table 2 and Fig. 3.

**Table 2:** Comparison results

**(a)**				
**Data**	**PINTnet**		**OGB**	
	**Evidence-supported** ^a^	**All** ^b^	**Evidence-supported** ^a^	**All** ^b^
Dataset1	9	92	1	109
Dataset2	15	122	2	268
Dataset3	1	149	1	291
**(b)**				
**Data**	**PINTnet**		**OGB**	
	**Evidence-supported** ^c^	**All** ^d^	**Evidence-supported** ^c^	**All** ^d^
Dataset1	9.500	3.067	3.800	2.344
Dataset2	9.700	3.697	4.000	3.829
Dataset3	5.000	2.922	6.000	4.376
**(c)**				
**Data**	**Found** ^e^		**All** ^f^	
	**PINTnet**	**OGB**		
Dataset1	8	6	8	
Dataset2	10	9	10	
Dataset3	6	4	6	

(a) The number of edges between the pathways in the pathway interaction network. The first column of each approach is the number of edges between the evidence-supported pathways. The second column of each approach is the number of edges between all pathways in the network

(b) The average degree of the pathways in the pathway interaction network. The first column of each approach is the average degree of the evidence-supported pathways. The second column of each approach is that of all pathways in the network

(c) The number of evidence-supported pathway found in the network. The first column is the number of evidence-supported pathways that are found in the pathway interaction network constructed using our method. The second column is the number of evidence-supported pathways that are found in the pathway interaction network constructed using OGB. The third column is the number of all evidence-supported pathways

^a^The number of edges connecting the evidence-supported pathways

^b^The total number of edges in the pathway interaction network

^c^The average degree of the evidence-supported pathways

^d^The average degree of all pathways in the pathway interaction network

^e^The number of evidence-supported pathways in the pathway interaction networks constructed using both approaches

^f^The total number of evidence-supported pathways

**Fig. 4:**
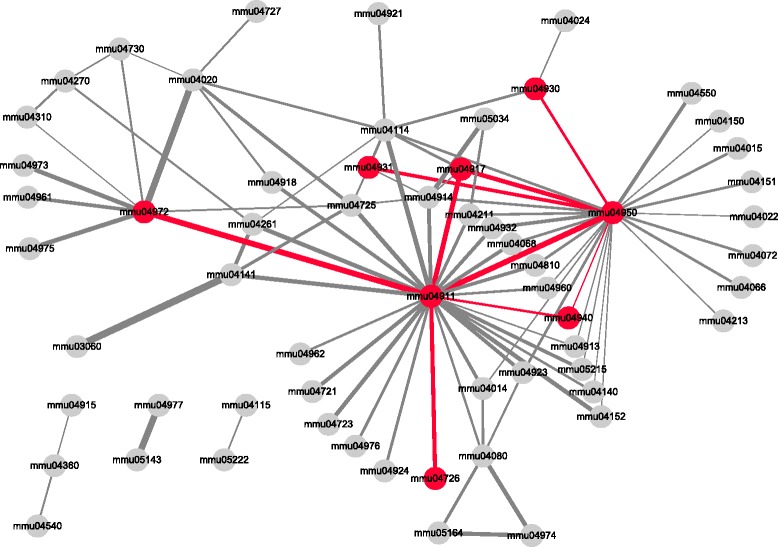
A pathway interaction network of pregnant mice. Sixty pathways are connected by 92 edges in this network. The pathways that coincide with the result of the original paper are rescued. The pathways are serotonergic synapse (mmu04726), insulin secretion (mmu04911), prolactin signaling pathway (mmu04917), pancreatic secretion (mmu04972), insulin resistance (mmu04931) and three diabetic pathways (mmu04930, mmu04940 and mmu04950) and colored in red. The edges connecting these pathways are also colored in red. The width of edges is set according to the activation score. The higher the activation score, the thicker the edge

In the pathway interaction network constructed using our method on *dataset1* with the cutoff value of 0.95, there were 60 pathways and 92 edges between the pathways. The network is shown in Fig. 4. Among the pathways, serotonergic synapse (mmu04726), insulin secretion (mmu04911), insulin resistance (mmu04931), prolactin signaling pathway (mmu04917), pancreatic secretion (mmu04972) and three diabetic pathways (mmu04930, mmu04940 and mmu04950) were included as nodes. In addition, it was observed that the edges in the network connected insulin secretion and serotonergic synapse, insulin secretion and pancreatic secretion, insulin secretion and prolactin signaling pathway, insulin resistance and diabetes-related pathways, and prolactin signaling pathway and diabetes-related pathways. The interactions between the pathways suggest how biological response occur during pregnancy by the cooperative work of relevant pathways. In addition, these interactions may give a point of view to conceive how the interactions of pathways drive the expansion of beta cell mass. The edges connected to diabetic pathways may imply the high chances of being diagnosed with gestational diabetes mellitus due to insulin resistance. Meanwhile, OGB failed to detect insulin resistance and one of the diabetic pathways even though there were 93 pathways and 109 edges. Also, there was only one edge connecting two remaining diabetic pathways.

**Fig. 5:**
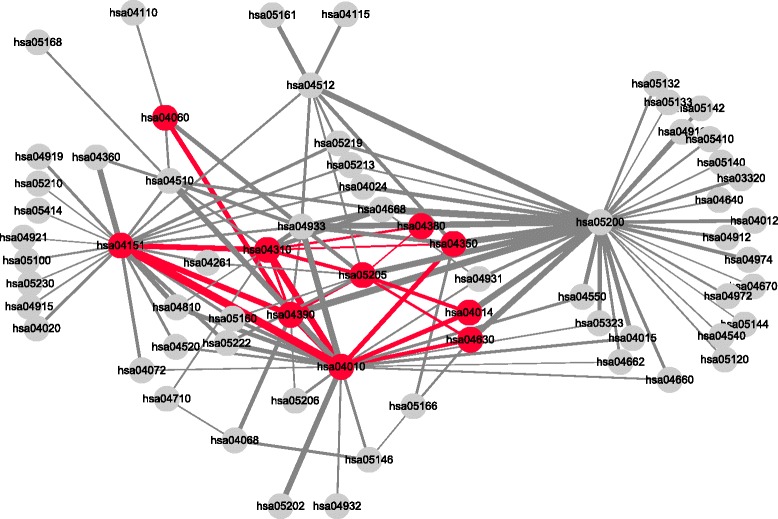
A pathway interaction network of bone metastasis from breast cancer. Sixty-six pathways are connected by 122 edges in this network. The original paper reported Jak-STAT signaling pathway (hsa04630), cytokine-cytokine receptor interaction (hsa04060), Hippo signaling pathway (hsa04390) and bone metastasis were upregulated in the control compared to ABL1/ABL2 knockdown mice. We found multiple paths from Ras signaling pathway (hsa04014), ABL kinases belong to, to osteoclast differentiation (hsa04380) through MAPK signaling pathway (hsa04010), Wnt signaling pathway (hsa04390), TGF- *β* signaling pathway (hsa04350), PI3K-Akt signaling pathway (hsa04151), Hippo signaling pathway (hsa04390) and proteoglycans in cancer (hsa05205). We found the evidences in literature that these pathways are related to bone metastasis from breast cancer. These pathways and the edges between the pathways are colored in red and the width of edges are set according to the activation score

There were 66 pathways and 122 edges between the pathways in the pathway interaction network constructed using our method on *dataset2* with the cutoff value of 0.99. The network is shown in Fig. 5. We observed Jak-STAT signaling pathway (hsa04630), Hippo signaling pathway (hsa04390), cytokine-cytokine receptor interaction (hsa04060) and osteoclast differentiation (hsa04380), which were reported by the original paper, were included in the network. In addition to the pathways, we identified the pathways that we found the evidences of functional importance in bone-metastatic breast cancer from the literatures lying on the multiple paths from Ras signaling pathway (hsa04014) to osteoclast differentiation. The pathways were MAPK signaling pathway (hsa04010), Wnt signaling pathway (hsa04310), Hippo signaling pathway, TGF- *β* signaling pathway (hsa04350), PI3K-Akt signaling pathway (hsa04151) and proteoglycans in cancer (hsa05205). The result implies that the pathways implicated in bone metastasis from breast cancer interact each other and the interactions among the pathways along with the paths may give the insight of how bone metastatic breast cancer is caused by pathways interaction. However, TGF- *β* signaling pathway was not rescued by OGB and only two edges were detected: MAPK signaling pathway and proteoglycans in cancer; Ras signaling pathway and PI3K-Akt signaling pathway.

**Fig. 6:**
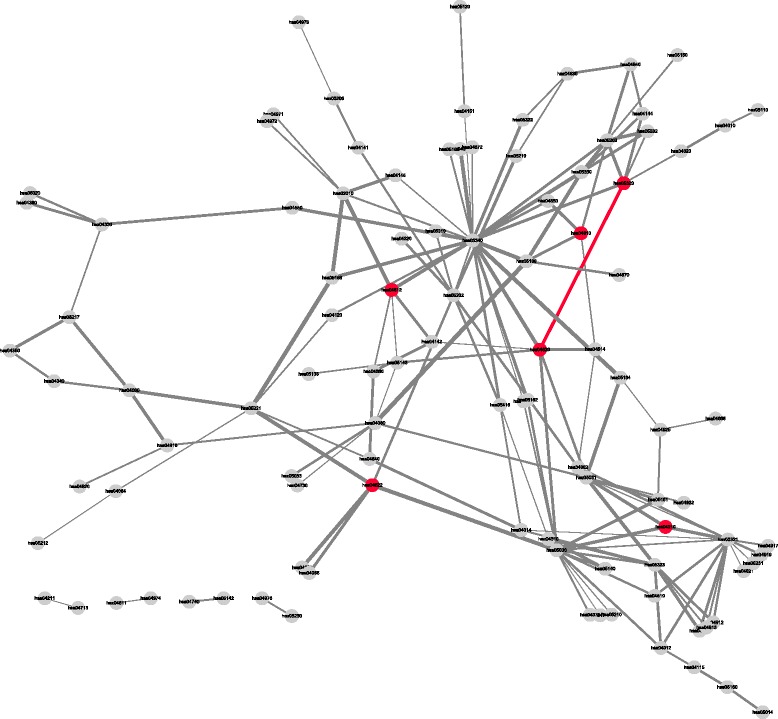
A pathway interaction network of IFN- *α* mediated autoimmunity. One hundred two pathways are connected by 149 edges in this network. The original paper reported that Toll-like receptor signaling pathway (hsa04620), complement and coagulation cascades (hsa04610), antigen processing and presentation (hsa04612), RIG-I-like receptor signaling pathway (hsa04622) and apoptosis (hsa04210) were upregulated and our method rescued the pathways including autoimmune thyroid disease (hsa05320). There is only one edge connecting these pathways and the edge connects Toll-like receptor signaling pathway and autoimmune thyroid disease

The pathway interaction network constructed using our method on *dataset3* with the cutoff value 0f 0.99 included 102 pathways and 149 edges between the pathways. The network is shown in Fig. 6. The network successfully included the pathways that mentioned to be upregulated in the original paper except RAR activation because there is no proper match of the pathway in KEGG. The pathways were Toll-like receptor signaling pathway (hsa04620), autoimmune thyroid disease (hsa05320), complement and coagulation cascades (hsa04610), antigen processing and presentation (hsa04612), apoptosis (hsa04210) and RIG-I-like receptor signaling pathway (hsa04622). There was only one edge between the pathways and Toll-like receptor signaling pathway and autoimmune thyroid disease were connected by the edge. However, Toll-like receptor signaling pathway is the pathway that IFN- *α* belongs to and autoimmune thyroid disease is the overall context of the original paper. Moreover, the findings reported in [60, 61] supported that Toll-like receptor signaling pathway plays an important role in autoimmunity as mentioned in Evaluation criteria section. On the contrary, OGB failed to detect RIG-I-like receptor signaling pathway and complement and coagulation cascades. In addition, there was only one edge between antigen processing and presentation and autoimmune thyroid disease.

## Discussion

Currently available approaches for constructing pathway network were designed to handle microarray data so the approaches mostly rely on the statistical tests. The approaches determine the significance of the interaction by the *p*-value yielded by the tests or use the *p*-value itself to calculate the secondary score for the determination. In addition, even though several approaches incorporated PPI to infer the interactions between pathways, the approaches have a limitation that PPI was treated merely as a set of individually represented genes without considering any relation between the genes. To address these issues, we applied the concept of closeness centrality and shortest paths to define the edges in the pathway interaction network. We assumed that the interaction between two pathways will occur when the biological signals rapidly flow through the topologically important genes. Based on the assumption, we constructed shortest path-weaved subnetworks to represent the edges and calculated interaction score using explicit gene expression quantity on the subnetworks.

The scoring scheme of PINTnet is a ranking method. It constructs the pathway interaction network using pairs of pathways of which the score is higher than the cutoff value. The results on the test datasets suggest that PINTnet successfully reproduced the results of the original papers and, therefore, is useful in analyzing the perturbed pathways and their interactions in a given condition.

Like existing methods, PINTnet is based on the identification of overlapping genes between two pathways. We assumed that the overlapping genes function as a bridge between two pathways. Based on the assumption, we considered the situation that at least one overlapping gene exists as one of the rules to define the edge in the pathway interaction network. This criterion, though reasonable and popular, may be too stringent. For example, when two pathways are well connected by direct edges in PPI but do not share any genes, it is not clear whether the two pathways interact or not. Therefore, the pairs of truly interacting pathways might be ruled out. We will further work on the matter to overcome the limitation.

## Conclusion

In this work, we developed a new pathway interaction network construction method, PINTnet. Running PINTnet on the three datasets to test the performance, we observed that it successfully rescued the findings reported in the original papers. In the result of *dataset1*, PINTnet successfully detected the pathways related to the changes occurring during pregnancy. Also we observed that the pathways were connected by the edges supported by the literatures. For *dataset2*, we also identified that the pathways related to bone-metastatic breast cancer were rescued in the pathway interaction network and the edges between the pathways implied the interactions participating in the induction of the phenotype. For *dataset3*, the pathways reported by the original paper were included as nodes in the pathway interaction network and there was a connected edge between Toll-like receptor signaling pathway and autoimmune thyroid disease. We expect PINTnet to be a useful tool for pathway interaction network analysis. PINTnet is available at http://biohealth.snu.ac.kr/software/PINTnet/.

## Abbreviations

AS: Activation score
CES: Co-expression score
DEG: Differentially expressed gene
GEO: Gene expression omnibus
GO: Gene ontology
GSEA: Gene set enrichment analysis
OGB: Overlapping gene-based approach
ORA: Over-representation analysis
PB: Protein-protein interaction-based approach
PPI: Protein-protein interaction
SPIA: Signaling pathway impact analysis
SSN: Shortest path-weaved subnetwork
